# Cancer mortality and morbidity among plutonium workers at the Sellafield plant of British Nuclear Fuels

**DOI:** 10.1038/sj.bjc.6690207

**Published:** 1999-03

**Authors:** R Z Omar, J A Barber, P G Smith

**Affiliations:** London School of Hygiene and Tropical Medicine, Keppel Street, London, WC1E 7HT, UK

**Keywords:** radiation, plutonium, nuclear workers, cohort, mortality, cancer incidence

## Abstract

The mortality of all 14 319 workers employed at the Sellafield plant of British Nuclear Fuels between 1947 and 1975 was studied up to the end of 1992, and cancer incidence was examined from 1971 to 1986, in relation to their exposures to plutonium and to external radiation. The cancer mortality rate was 5% lower than that of England and Wales and 3% less than that of Cumbria. The significant excesses of deaths from cancer of the pleura and thyroid found in an earlier study persist with further follow-up (14 observed, 4.0 expected for pleura; 6 observed, 2.2 expected for thyroid). All of the deaths from pleural cancer were among radiation workers. For neither site was there a significant association between the risk of the cancer and accumulated radiation dose. There were significant deficits of deaths from cancers of mouth and pharynx, liver and gall bladder, and larynx and leukaemia when compared with the national rates. Among all radiation workers, there was a significant positive association between accumulated external radiation dose and mortality from cancers of ill-defined and secondary sites (10-year lag, *P* = 0.04), leukaemia (no lag, *P* = 0.03; 2-year lag, *P* = 0.05), multiple myeloma (20-year lag, *P* = 0.02), all lymphatic and haematopoietic cancers (20-year lag, *P* = 0.03) and all causes of death combined (20-year lag, *P* = 0.008). Among plutonium workers, there were significant excesses of deaths from cancer of the breast (6 observed, 2.6 expected) and ill-defined and secondary cancers (29 observed, 20.1 expected). No significant positive trends were observed between the risk of deaths from cancers of any specific site, or all cancers combined, and cumulative plutonium and external radiation doses. For no cancer site was there a significant excess of cancer registrations compared with rates for England and Wales. Analysis of trends in cancer incidence showed significant increases in risk with cumulative plutonium plus external radiation doses for all lymphatic and haematopoietic neoplasms for 0-, 10- and 20-year lag periods. Taken as a whole, our findings do not suggest that workers at Sellafield who have been exposed to plutonium are at an overall significantly increased risk of cancer compared with other radiation workers. © 1999 Cancer Research Campaign


*British Journal of Cancer* (1999) **79**(7/8), 1288–1301
© 1999 Cancer Research Campaign
Article no. bjoc.1998.0207

### Cancer mortality and morbidity among plutonium workers at the Sellafield plant of British Nuclear Fuels

**RZ Omar*, JA Barber* and PG Smith**

London School of Hygiene and Tropical Medicine, Keppel Street, London WC1E 7HT, UK

**Summary** The mortality of all 14 319 workers employed at the Sellafield plant of British Nuclear Fuels between 1947 and 1975 was studied up to the end of 1992, and cancer incidence was examined from 1971 to 1986, in relation to their exposures to plutonium and to external radiation. The cancer mortality rate was 5% lower than that of England and Wales and 3% less than that of Cumbria. The significant excesses of deaths from cancer of the pleura and thyroid found in an earlier study persist with further follow-up (14 observed, 4.0 expected for pleura; 6 observed, 2.2 expected for thyroid). All of the deaths from pleural cancer were among radiation workers. For neither site was there a significant association between the risk of the cancer and accumulated radiation dose. There were significant deficits of deaths from cancers of mouth and pharynx, liver and gall bladder, and larynx and leukaemia when compared with the national rates. Among all radiation workers, there was a significant positive association between accumulated external radiation dose and mortality from cancers of ill-defined and secondary sites (10-year lag, $P$ = 0.04), leukaemia (no lag, $P$ = 0.03; 2-year lag, $P$ = 0.05), multiple myeloma (20-year lag, $P$ = 0.02), all lymphatic and haematopoietic cancers (20-year lag, $P$ = 0.03) and all causes of death combined (20-year lag, $P$ = 0.008). Among plutonium workers, there were significant excesses of deaths from cancer of the breast (6 observed, 2.6 expected) and ill-defined and secondary cancers (29 observed, 20.1 expected). No significant positive trends were observed between the risk of deaths from cancers of any specific site, or all cancers combined, and cumulative plutonium and external radiation doses. For no cancer site was there a significant excess of cancer registrations compared with rates for England and Wales. Analysis of trends in cancer incidence showed significant increases in risk with cumulative plutonium plus external radiation doses for all lymphatic and haematopoietic neoplasms for 0-, 10- and 20-year lag periods. Taken as a whole, our findings do not suggest that workers at Sellafield who have been exposed to plutonium are at an overall significantly increased risk of cancer compared with other radiation workers.

**Keywords:** radiation; plutonium; nuclear workers; cohort; mortality; cancer incidence

Workers in nuclear plants are at potential risk of exposure to ionizing radiations, both externally from radioactivity in the working environment and internally from radionuclides which enter the body by inhalation, ingestion or through accidents resulting in percutaneous wounds. The possible carcinogenic effects of exposure to external sources of radiation among nuclear workers have been the subject of a large number of studies (Kendall et al, 1992; Gilbert et al, 1993; Carpenter et al, 1994; Cardis et al, 1995), but there have been few studies of internal exposure to radionuclides among these groups. We have reported previously on mortality and cancer morbidity among workers at the Sellafield plant of British Nuclear Fuels (BNFL) (Smith and Douglas, 1986; Douglas et al, 1994). We examined possible risks associated with exposure to external radiation, but at these times the data available on exposure to radionuclides were inadequate for detailed analysis. Among workers at the Sellafield plant, the element of most concern is plutonium. More workers are potentially exposed to the isotopes of plutonium than other radionuclides and the contribution of plutonium exposure to the collective internal dose is far greater than that of other radionuclides (Riddell et al, 1998).

*Received 18 May 1998*
*Accepted 27 May 1998*

*Correspondence to:* PG Smith

Exposure to external radiation involves relatively uniform exposure of a worker to sparsely ionizing, penetrating radiation. Plutonium emits alpha particles which traverse only a few cells before coming to a halt. It is a biological hazard, therefore, when taken into the body, in which the alpha particles irradiate those cells close to where the plutonium is deposited. Plutonium is physically long-lived and also is retained in the body with a long biological half-life, so that it delivers its dose to adjacent tissues over a long period of time. The biochemistry of plutonium is such that it is generally concentrated in particular parts of the body and this leads to a heterogeneous distribution of the dose from plutonium. Plutonium concentrates, principally, in the liver and in the skeleton, so that the risk of cancer through exposure to plutonium is likely to be greatest for the lung (if inhaled), liver, bone and, possibly, leukaemia. Densely ionizing alpha particles cause more biological damage than sparsely ionizing radiations and a weighting factor of 20 is applied to the absorbed doses to generate equivalent doses (Committee on Biological Effects of Ionizing Radiations, 1988; Clarke et al, 1996).

The Sellafield plant, on the Cumbrian coast in the UK, was developed originally for the production of plutonium for nuclear weapons. Later, plutonium was produced as a consequence of the commercial reprocessing of spent nuclear fuel and has been stored on the site. Workers have been monitored for possible exposure to

**Present address:* Department of Medical Statistics and Evaluation, Imperial College School of Medicine, Du Cane Road, London W12 0NN, UK.

**Table 1** Study population at 1 January 1993

| | Men (%) | Women (%) | Total (%) |
|---|---|---|---|
| Total number of workers | 11 679 (100.0) | 2689 (100.0) | 14 385 (100.0) |
| No. for whom date of birth not known | 44 (0.4) | 5 (0.2) | 66[a] (0.5) |
| No. eligible for analysis | 11 635 (99.6) | 2684 (99.8) | 14 319 (99.5) |
| Alive 1 January 1993 | 7 800 (66.8) | 2137 (79.5) | 9937 (69.1) |
| Died before 1993 | 3411 (29.2) | 445 (16.5) | 3854 (26.8) |
| Emigrated | 401 (3.4) | 90 (3.3) | 491 (3.4) |
| Untraced | 23 (0.2) | 12 (0.5) | 35 (0.2) |
| Total years of follow-up | 332 532.4 | 82 899.3 | 415 431.6 |
| Average duration of follow-up (years) | 28.6 | 30.9 | 29.0 |

[a]Includes 17 of unknown sex.

plutonium since it was first produced in the plant. The most likely route of exposure has been through inhalation, and monitoring has been conducted principally through the analysis of urine samples collected from workers. These monitoring data have been assembled comprehensively by BNFL and estimates have been made of plutonium uptake for individual workers for each year during which they may have been exposed. These uptake estimates have been used further to estimate annual radiation doses to specific organs where plutonium might be deposited (Riddell et al, 1998). We have related these dose estimates to cancer mortality and morbidity in the work force since construction of the plant started in 1947.

**Table 2** Estimated cumulative collective radiation doses from external radiation and from plutonium exposure

External radiation

| | Total cumulative dose (mSv) |
|---|---|
| All radiation workers ($n$ = 10 382) | 1 352 326 |
| All plutonium workers ($n$ = 5203) | 1 023 534 |
| Plutonium workers with plutonium dose assessed ($n$ = 4609) | 958 868 |

Plutonium exposure

| | Cumulative organ dose (mSv)[b] | |
|---|---|---|
| Organ | 'W' | 'Y' |
| Bone surfaces | 3 282 100 | |
| Lungs | 44 576 | 896 177 |
| Red bone marrow | 265 635 | |
| Small intestines[a] | 8047 | 8052 |
| Lower large intestines[a] | 8107 | 8232 |
| Upper large intestines[a] | 8063 | 8099 |
| Stomach[a] | 8045 | 8047 |
| Liver | 420 872 | |
| Soft tissues | 8045 | |
| Testes | 39 685 | |
| 'Effective' whole body (see text) | 219 099 | 355 369 |

[a]The major contribution to the dose to these organs is composed of the estimated 'soft tissues' dose. [b]Different organ doses assessed according to the assumed solubility class ('W' or 'Y') of plutonium compounds to which there was exposure (see text).

### POPULATION AND METHODS

We have described previously the study population, the nature and source of external radiation exposure data and the methods used to determine the vital status, causes of death and cancer registrations (Smith and Douglas, 1986; Douglas et al, 1994). We summarize this information briefly below, together with the details of plutonium exposure and the methods used to derive estimates of plutonium uptake and organ-specific doses.

### Study population

The study population consisted of all workers (14 385) employed by BNFL or its predecessors at the Sellafield plant at any time between the date it opened, in 1947, and 1 January 1976. In our previous paper, the number of such workers was given as 14 282 (Douglas et al, 1994). Subsequent investigations made by BNFL staff, using personnel and plant medical records, resulted in the exclusion of six persons who were working at another BNFL plant and one who had been incorrectly included in the study twice. Records of a further 110 workers were also discovered who should have been included in the study population. Most of these were employed during the early years of the plant's operation.

### Follow-up

For the analyses reported in this paper, follow-up of the study population was extended from 1 January 1989 (Douglas et al, 1994) to 1 January 1993. For all workers whose dates of birth and sex were known, an attempt was made to trace their vital status through the National Health Service Central Registers (NHSCRs). Information on cancer registrations was derived from the same source. Of the 14 319 (99.5%) workers with known date of birth and sex, 9937 (69.4%) were traced as alive on 1 January 1993, 3854 (26.9%) had died, 491 (3.4%) were known to have emigrated and 35 (0.2%) could not be traced (Table 1). Workers in the last two groups were included in the analyses up to their dates of emigration or last employment at the plant, respectively. Death certificates were obtained for each worker who had died and the underlying cause of death recorded was coded according to the International Classification of Diseases and Causes of Death (ICD) in operation at the time of death.

The coverage of the national cancer registration scheme was reasonably complete only by 1971 and, therefore, analyses of

© Cancer Research Campaign 1999

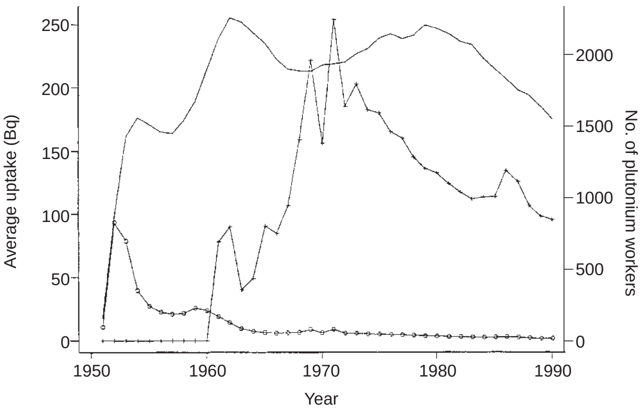

**Figure 1** Numbers of plutonium workers employed by year and average annual uptakes of $^{241}Pu$ and plutonium alpha among workers for whom assessments were made. ∘, average plutonium alpha, +, average $^{241}Pu$; –, no. of plutonium workers

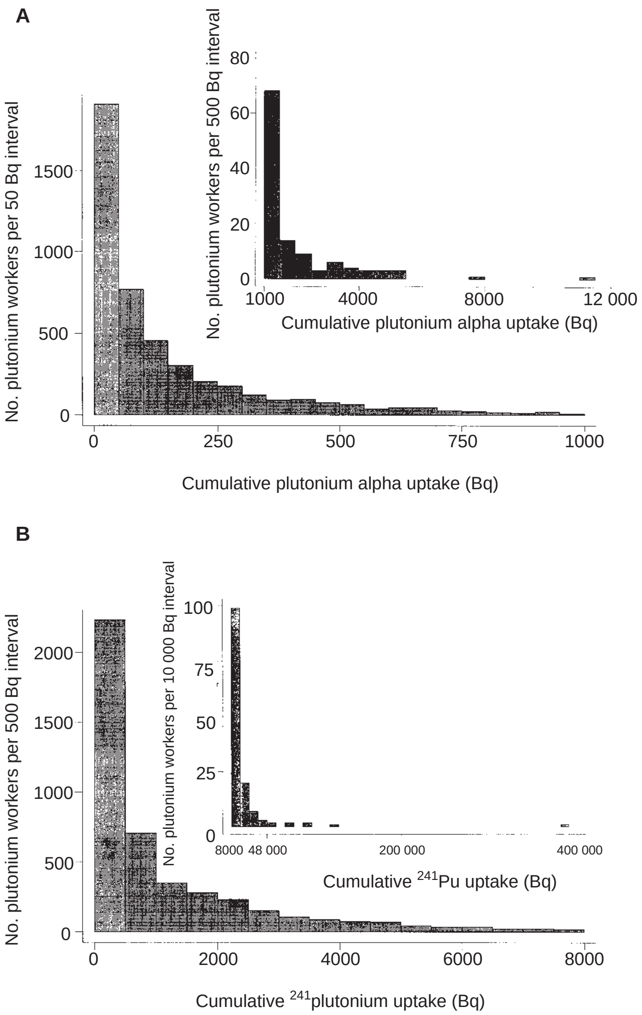

**Figure 2** Cumulative estimated uptakes of (**A**) plutonium alpha and (**B**) $^{241}Pu$ among plutonium workers for whom uptakes were estimated cancer registrations were restricted to those workers known to be alive on 1 January 1971 or who joined the work force after that date. Within the study population, a comparison was made, by year, of the proportion of deaths from cancer for which a cancer had also been registered in the national scheme. This showed a similar pattern to that reported previously (Douglas et al, 1994), with a decline in the percentages of cancer deaths with previous cancer registrations from 1987 onwards. We have restricted analysis of cancer registrations to the period from 1971 to 1986 (inclusive). Of the 13 206 workers eligible for inclusion in analyses of cancer incidence, 664 were registered with a cancer (other than of the skin, which site was excluded from analyses because of the known poor registration of such cancers).

### Comparative death and cancer incidence rates

Age, sex and cause-specific death rates for the population of England and Wales were obtained from the Office for National Statistics (ONS) for each of the years 1950–92 (in 5-year age groups up to the age of 85 years and for 85 years and older). Cancer registration rates were obtained for both England and Wales (1971–86) and for the Northern Region (1971–84). Professor M Gardner and Dr P Winter had previously provided us with mortality rates from selected causes for Cumberland for 1968–78 (Gardner et al, 1983, 1984) which we used to estimate death rates up to 1978. Data for the period 1980–92 from ONS were used to estimate Cumbrian death rates from 1979 to 1992. Cumbria is the county in which the Sellafield plant is located and it overlaps substantially with the old county of Cumberland which existed before boundary and name changes in 1974.

### Radiation doses

### *External radiation*

The methods used to assess and validate external exposure to radiation have been given previously (Smith and Douglas, 1986). Briefly, records of radiation doses, estimated from film badge dosimeters worn on the trunk, have been kept by BNFL for all workers who, other than infrequently, entered areas where it was possible they would be exposed to radiation (‘controlled areas’). The 10 382 workers for whom such records were kept, or who had been monitored for internal radiation exposure (see below), have been designated ‘radiation workers’ and all other workers as ‘non-radiation workers’. For each radiation worker, BNFL supplied us with an annual dose of whole-body penetrating radiation (in mSv), for each of the years 1947–90 (Douglas et al, 1994).

### *Plutonium exposure*

There are two types of plutonium exposure recorded at Sellafield. These are ‘plutonium alpha’ radioisotopes of plutonium (mostly $^{239}Pu$ but some $^{240}Pu$ and $^{238}Pu$) that decay via the emission of alpha particles, and $^{241}Pu$ which decays by beta particle emission to the alpha emitter americium 241. A proportion of the plutonium which enters into the body after exposure is excreted in urine and the amount excreted can be used to estimate the quantity of plutonium retained in the body. This is expressed as activity taken in and retained per year and is termed the plutonium uptake.

Riddell et al (1998) detail the methods used by BNFL to assess the radiation doses acquired by workers at the Sellafield plant through exposure to plutonium. A summary of the approach adopted is given below.

 *© Cancer Research Campaign 1999*

**Table 3** Standardized mortality ratios (SMRs) for all causes of death and all cancers among plutonium workers, other radiation workers and all workers, comparing rates with those for England and Wales (E&W) and Cumbria

| Group of workers | All cancers: No. of deaths | All cancers: SMR E&W | All cancers: SMR Cumbria | Causes other than cancer: No. of deaths | Causes other than cancer: SMR E&W | Causes other than cancer: SMR Cumbria | All causes: No. of deaths | All causes: SMR E&W | All causes: SMR Cumbria |
|---|---|---|---|---|---|---|---|---|---|
| Radiation | 738 | 96 | 97 | 1944 | 97 | 89*** | 2682 | 97* | 91*** |
| Plutonium | 384 | 100 | 101 | 961 | 97 | 90*** | 1345 | 98 | 92** |
| Other radiation | 354 | 92 | 93 | 983 | 97 | 89*** | 1337 | 96 | 90*** |
| Non-radiation | 299 | 93 | 96 | 873 | 102 | 94* | 1172 | 100 | 94* |
| All | 1037 | 95* | 97 | 2817 | 99 | 91** | 3854 | 98 | 92*** |

$^{*}P < 0.05$; $^{**}P < 0.01$; $^{***}P < 0.001$.

Assessment of plutonium uptake by Sellafield workers has been made, principally, through monitoring urine samples. Urine samples have been collected on a regular basis for persons working in areas of the plant where such exposure might occur. We have classified the 5203* workers who were ever monitored for plutonium exposure as ‘plutonium workers’ (irrespective of whether or not plutonium was ever detected in a urine sample). For 43 of these workers, we were not supplied with a film badge record, but for purposes of analysis they have been classified as radiation workers. The frequency of urine collections and the methods for assessing the samples have changed over time, as more sensitive detection methods were developed.

Britcher et al (1994) have summarized plutonium monitoring procedures at Sellafield at different times. The first records of urine monitoring for plutonium exposure in the plant date from 1951. This was shortly after the start of reprocessing operations, when a pilot monitoring programme was instituted. By 1952, a routine monitoring scheme was operating for persons working in areas where plutonium exposure was possible. Urine samples were collected from workers in those areas at 3- or 6-month intervals. Additional samples were collected if an excretion rate of 46 mBq or more was measured in a sample. The volume of urine that was required to be collected was only 100 ml. In 1961, the frequency of urine sampling was increased to monthly for those working full-time in areas where exposure was possible, and bimonthly for those who worked for only some of the time in such areas. Additional urine samples were collected from workers with abnormal routine samples [e.g. those with activity not consistent with a previous result or with the general level (A Riddell, personal communication)]. In 1968, the urine sampling procedure was modified to collect five 1 l samples at six-month intervals. An additional three 1 l samples were collected after potential acute exposure events. Since 1980, four 1 l samples have been collected at 6-month intervals for routine monitoring of workers. The assay methods have improved over time. Before 1961, the minimum detectable plutonium level in a urine sample was 4.6 mBq [though levels were generally reported only as ‘less than 46 mBq’, which was a local control limit that was applied in the plant, with higher values leading to specific investigations (Dr R Strong, personal communication)]. In 1961, this reduced to 2.3 mBq and in 1986 to 0.5 mBq (Britcher et al, 1994).

### *Assessment of plutonium uptake*

Two methods have been used by BNFL for assessing the uptake of plutonium alpha by individual workers. Individual assessments of uptake (‘special assessments’) were made for 993 workers for a variety of reasons, including those known to have been involved in an exposure ‘incident’ or who had claimed compensation for cancers potentially caused by radiation exposures incurred at the plant. These assessments involved a detailed consideration by a health physicist of likely exposures, based on full work history records and the circumstances of known acute exposure incidents as well as on the urine assays. ‘Standardized’ assessments were made for 3616 workers by assuming that exposure to plutonium started 6 months before the date on which the first urine sample was collected for a worker and ended at the date of the last urine sample. For purposes of estimation, uptake was assumed to be at a constant rate between these dates. Annual uptakes of plutonium alpha were estimated from the assays on urine samples based on a mathematical function, which was derived assuming decay according to an exponential function in the amounts excreted in urine with time since exposure (Jones, 1985).

A study conducted in the late 1960s revealed substantial adventitious contamination of urine samples. This led to changes in sampling arrangements, which were in place by 1971 (Britcher et al, 1994). Therefore, for workers for whom urine samples had been collected in 1971 or later, only the post 1970 samples were used for the estimation of uptake (including for the period before 1971 by extrapolation) and results from earlier urine samples were ignored. Such assessments were possible for 1629 (45%) of the 3616 workers having standardized assessments of uptake. Uptakes for the other 1987 workers for whom standardized assessments were made were based on the pre-1971 data. For 725 of the workers who had provided a urine sample after 1960, of whom 579 had provided at least one sample after 1970, no sample had given results above the minimum level of plutonium that could be detected. An upper limit uptake assessment was generated for these workers by setting the last sample result equal to the relevant limit of detection.

No assessments were made for 585 workers who had only provided urine samples before 1961, and all of which were recorded as less than 46 mBq. In addition, uptake assessments were not available for nine plutonium workers who had no usable urine data. These 594 workers were known to have been potentially exposed to plutonium, however, and are classified as plutonium workers in our analyses.

Radiochemical analysis of urine samples was not carried out for the assessment of $^{241}$Pu and, hence, ingrown americium-241.

*The number of plutonium workers (5203) is slightly smaller than that given in Riddell et al (1998) (5250) as for some workers there were inconsistencies in the data, in particular the dates of monitoring for plutonium fell outside their recorded employment period.

Uptake of $^{241}Pu$ was inferred from the assessed plutonium alpha uptake by the application of a factor relating $^{241}Pu$ to plutonium alpha in plant material on a yearly basis depending upon the average recorded burn-up of the fuel reprocessed in each year (Riddell and Britcher, 1994). The Sellafield plant was mostly engaged in military production before 1961 and there was very little exposure to $^{241}Pu$ during this period.

It has not been possible to validate satisfactorily estimates of plutonium uptake based upon the measurement of plutonium alpha excreted in urine, derived using the Jones model (Jones, 1985). However, assessments of plutonium burden in the small number of workers who had a detailed autopsy have indicated that the Jones model, on average, overestimates the body burden by around a factor of three (Lawson et al, 1989; Riddell et al, 1998). The estimates of doses based on uptake of plutonium based on the Jones model had been reduced by a factor of three to allow for this overestimation, before they were provided to us by BNFL.

### *Organ doses*

The doses to each organ in each year were calculated by BNFL using dosimetric models based on the distribution of plutonium activity between organs and its clearance using standard metabolic models recommended by the International Commission on Radiological Protection (ICRP, 1986). These models relate to a reference 70-kg male worker and, thus, only approximate the deposition, distribution and retention of radionuclides in any given individual. Estimates of organ-specific doses (in mSv) were made for small intestines, upper and lower large intestines, stomach, lung, soft tissues, liver, testes, bone and red bone marrow. Doses inferred from urine samples as having been received in the lung, stomach, upper and lower large intestines and small intestines depend on the chemical solubility of the plutonium. The plutonium present at Sellafield has been classified as 'W' or 'Y' (weeks or years), which refer to its retention time in the lungs, depending on the solubility class of the plutonium. This classification applies to

**Table 4** Observed and expected numbers of deaths from cancers of specific sites among plutonium, other radiation and non-radiation workers and rate ratios comparing radiation workers with non-radiation workers and plutonium workers to other radiation workers

| Cancer site (ICD code – eighth revision) | Plutonium workers | | | Other radiation workers | | | Rate ratio (plutonium vs. other rad.) | Non-radiation workers | | | Rate ratio (rad. vs non-rad) | All workers | | | |
|---|---|---|---|---|---|---|---|---|---|---|---|---|---|---|---|
| | Obs | Exp E&W | SMR E&W | Obs | Exp E&W | SMR E&W | | Obs | Exp E&W | SMR E&W | | Obs | Exp E&W | SMR E&W | SMR Cumbria |
| Lip (140) | 0 | 0.14 | 0 | 1 | 0.15 | 680 | 0.00 | 0 | 0.12 | 0 | ∞ | 1 | 0.40 | 247 | |
| Tongue (141) | 1 | 1.29 | 77 | 2 | 1.28 | 156 | 0.47 | 0 | 0.94 | 0 | ∞ | 3 | 3.52 | 85 | |
| Mouth and pharynx (143–149) | 0 | 3.97 | 0 | 3 | 3.91 | 77 | 0.00 | 3 | 2.88 | 104 | 0.24 | 6 | 10.76 | 56* | |
| Oesophagus (150) | 16 | 13.35 | 120 | 14 | 12.74 | 110 | 0.96 | 8 | 9.05 | 88 | 1.92 | 38 | 35.15 | 108 | 104 |
| Stomach (151) | 33 | 34.43 | 96 | 45 | 34.71 | 130 | 0.72 | 32 | 27.48 | 116 | 0.93 | 110 | 96.66 | 114 | 103 |
| Small intestines (152) | 2 | 0.76 | 263 | 0 | 0.77 | 0 | ∞ | 0 | 0.62 | 0 | ∞ | 2 | 2.15 | 93 | |
| Colon (153) | 25 | 24.52 | 102 | 27 | 24.64 | 110 | 0.89 | 20 | 22.68 | 88 | 0.92 | 72 | 71.86 | 100 | 92 |
| Rectum (154) | 17 | 16.91 | 101 | 15 | 16.83 | 89 | 1.11 | 20 | 13.70 | 146 | 0.62 | 52 | 47.45 | 110 | 111 |
| Liver and gall bladder (155–156) | 1 | 5.17 | 19** | 1 | 5.08 | 20** | 0.85 | 2 | 4.14 | 48 | 0.34 | 4 | 14.40 | 28*** | |
| Pancreas (157) | 17 | 16.32 | 104 | 15 | 16.07 | 93 | 1.00 | 12 | 12.92 | 93 | 1.13 | 44 | 45.32 | 97 | 88 |
| Larynx (161) | 3 | 3.65 | 82 | 0 | 3.55 | 0 | ∞ | 2 | 2.34 | 86 | 0.74 | 5 | 9.54 | 52* | |
| Lung (162) | 133 | 145.78 | 91 | 113 | 141.09 | 80** | 1.12 | 92 | 94.37 | 97 | 0.98 | 338 | 381.35 | 89* | 96 |
| Pleura (163.0) | 8 | 1.70 | 471*** | 6 | 1.54 | 390* | 1.15 | 0 | 0.76 | 0 | ∞* | 14 | 3.99 | 351*** | |
| Bone (170) | 0 | 1.10 | 0 | 1 | 1.23 | 81 | 0.00 | 1 | 1.02 | 98 | 1.12 | 2 | 3.34 | 60 | |
| Connective tissue (171) | 0 | 1.31 | 0 | 3 | 1.33 | 226 | 0.00 | 0 | 1.02 | 0 | ∞ | 3 | 3.66 | 82 | |
| Melanoma (172) | 1 | 2.74 | 36 | 4 | 2.84 | 141 | 0.24 | 3 | 2.28 | 131 | 0.65 | 8 | 7.87 | 102 | |
| Breast (174) | 6 | 2.55 | 236* | 2 | 5.92 | 34* | 7.66** | 21 | 28.35 | 74 | 1.35 | 29 | 36.83 | 79 | 97 |
| Uterus (180–182) | 0 | 0.55 | 0 | 2 | 1.48 | 135 | 0.00 | 9 | 8.28 | 109 | 1.46 | 11 | 10.32 | 107 | 111 |
| Ovary (183) | 1 | 0.63 | 158 | 1 | 1.65 | 60 | 2.18 | 7 | 8.93 | 78 | 1.92 | 9 | 11.22 | 80 | 88 |
| Other female genital (184) | 0 | 0.04 | 0 | 0 | 0.11 | 0 | – | 1 | 0.74 | 134 | – | 1 | 0.89 | 112 | |
| Prostate (185) | 22 | 21.36 | 103 | 22 | 20.62 | 107 | 0.95 | 7 | 13.32 | 53* | 2.21* | 51 | 55.30 | 92 | 95 |
| Testis (186) | 2 | 1.39 | 144 | 2 | 1.62 | 123 | 1.51 | 1 | 0.73 | 138 | 1.32 | 5 | 3.74 | 134 | |
| Other male genital (172.5, 187) | 0 | 0.58 | 0 | 0 | 0.58 | 0 | – | 0 | 0.34 | 0 | – | 0 | 1.50 | 0 | |
| Bladder (188) | 17 | 13.86 | 123 | 12 | 13.58 | 88 | 1.25 | 10 | 9.93 | 101 | 0.88 | 39 | 37.37 | 104 | 108 |
| Kidney (189) | 5 | 7.28 | 69 | 8 | 7.07 | 113 | 0.56 | 2 | 4.85 | 41* | 2.71 | 15 | 19.21 | 78 | 73 |
| Brain and CNS (191–192) | 10 | 10.46 | 96 | 8 | 10.57 | 76 | 1.35 | 6 | 7.39 | 81 | 0.87 | 24 | 28.44 | 84 | |
| Thyroid (193) | 1 | 0.67 | 150 | 3 | 0.70 | 429* | 0.28 | 2 | 0.79 | 253 | 2.45 | 6 | 2.16 | 278* | |
| Ill-defined and secondary (195–199) | 29 | 20.13 | 144* | 16 | 19.52 | 82 | 1.90* | 23 | 16.13 | 143 | 0.96 | 68 | 55.78 | 122 | |
| Non-Hodgkin's lymphoma (200, 202) | 12 | 8.18 | 147 | 8 | 8.13 | 98 | 1.39 | 2 | 6.00 | 33* | 2.83 | 22 | 22.32 | 99 | |
| Hodgkin's disease (201) | 4 | 2.95 | 136 | 1 | 3.28 | 30* | 3.71 | 3 | 2.28 | 132 | 0.54 | 8 | 8.50 | 94 | |
| Multiple myeloma (203) | 5 | 4.63 | 108 | 3 | 4.49 | 67 | 1.67 | 3 | 3.59 | 84 | 0.88 | 11 | 12.71 | 87 | |
| Leukaemia (204–208) | 7 | 9.86 | 71 | 9 | 10.17 | 88 | 0.79 | 4 | 8.10 | 49* | 1.27 | 20 | 28.14 | 71* | 75 |
| Other neoplasms (140–209 excluding above) | 6 | 7.82 | 77 | 7 | 6.79 | 103 | 0.92 | 3 | 5.38 | 56 | 1.86 | 16 | 19.06 | 84 | |
| All neoplasms | 384 | 385.10 | 100 | 354 | 384.04 | 92 | 1.05 | 299 | 321.45 | 93 | 1.06 | 1037 | 1090.91 | 95* | 97 |
| Person–years at risk | 134 817.2 | | | 154 552.5 | | | | 126 061.9 | | | | 415 431.6 | | | |

**P* < 0.05; ***P* < 0.01; ****P* < 0.001.

 *© Cancer Research Campaign 1999*

**Table 5** Observed and expected numbers of deaths from causes other than cancers among plutonium, other radiation and non-radiation workers and rate ratios comparing radiation workers with non-radiation workers and plutonium workers with other radiation workers

| Cause of death (ICD code – eighth revision) | Plutonium workers | | | Other radiation workers | | | Rate ratio (plutonium vs. other rad.) | Non-radiation workers | | | Rate ratio (rad. vs non-rad) | All workers | | | |
|---|---|---|---|---|---|---|---|---|---|---|---|---|---|---|---|
| | Obs | Exp E&W | SMR E&W | Obs | Exp E&W | SMR E&W | | Obs | Exp E&W | SMR E&W | | Obs | Exp E&W | SMR E&W | SMR Cumbria |
| Infectious and parasitic (001–136) | 5 | 10.75 | 47* | 8 | 12.20 | 66 | 0.63 | 11 | 13.07 | 84 | 0.63 | 24 | 36.07 | 67* | 102 |
| Tuberculosis (100–199) | 0 | 6.54 | 0 | 2 | 7.60 | 26** | 0.00 | 9 | 8.77 | 103 | 0.18* | 11 | 22.94 | 48** | 59* |
| Benign and unspecified neoplasms (210–239) | 2 | 4.00 | 49 | 5 | 4.04 | 118 | 0.38 | 4 | 3.92 | 102 | 0.37 | 11 | 12.21 | 90 | |
| Endocrine, nutrition and metabolic disorders and immunity disorders (240–279) | 13 | 15.32 | 85 | 10 | 15.56 | 64 | 1.42 | 12 | 14.46 | 83 | 0.72 | 35 | 45.35 | 77 | |
| Diabetes (250) | 10 | 11.82 | 85 | 8 | 11.90 | 67 | 1.24 | 9 | 10.88 | 83 | 0.70 | 27 | 34.61 | 78 | 72* |
| Diseases of blood and blood-forming organs (280–289) | 1 | 2.23 | 45 | 3 | 2.44 | 123 | 0.34 | 4 | 2.76 | 145 | 0.43 | 8 | 7.43 | 108 | |
| Aplastic anaemia (284) | 0 | 0.54 | 0 | 1 | 0.57 | 175 | 0.00 | 2 | 0.55 | 366 | 0.60 | 3 | 1.65 | 181 | |
| Other anaemias (280–283, 285) | 1 | 0.96 | 104 | 1 | 1.11 | 90 | 0.90 | 1 | 1.51 | 66 | 0.64 | 3 | 3.58 | 84 | |
| Other blood disorders (286–289) | 0 | 0.74 | 0 | 1 | 0.78 | 128 | 0.00 | 1 | 0.71 | 140 | 0.17 | 2 | 2.23 | 90 | |
| Mental disorders (290–315) | 10 | 7.23 | 138 | 7 | 7.73 | 91 | 1.37 | 11 | 8.55 | 129 | 0.63 | 28 | 23.51 | 119 | |
| Diseases of the nervous and sense organs (320–389) | 11 | 19.25 | 57* | 18 | 20.09 | 90 | 0.59 | 11 | 17.59 | 63* | 1.18 | 40 | 56.95 | 70** | |
| Epilepsy (345) | 0 | 2.47 | 0 | 0 | 2.81 | 0 | – | 0 | 2.08 | 0 | – | 0 | 5.28 | 0 | |
| Multiple sclerosis (340) | 2 | 2.77 | 72 | 5 | 2.96 | 169 | 0.36 | 0 | 2.94 | 0 | ∞ | 7 | 8.68 | 81 | |
| Disease of circulatory system (390–458) | 723 | 656.90 | 110** | 690 | 658.70 | 105 | 1.05 | 576 | 542.18 | 106 | 1.01 | 1989 | 1858.19 | 107** | 94** |
| Ischaemic heart disease (410–414) | 498 | 451.47 | 110* | 485 | 442.96 | 109* | 1.01 | 371 | 322.98 | 115** | 0.96 | 1354 | 1217.67 | 111*** | 97 |
| Cerebrovascular diseases (430–438) | 137 | 108.21 | 127** | 117 | 114.11 | 103 | 1.27* | 111 | 119.51 | 93 | 1.28* | 365 | 341.91 | 107 | 89* |
| Diseases of respiratory system (460–519) | 99 | 142.33 | 70*** | 111 | 150.24 | 74*** | 0.88 | 121 | 138.23 | 88 | 0.78* | 331 | 430.93 | 77*** | 82*** |
| Influenza (470–474) | 4 | 2.83 | 141 | 4 | 3.39 | 118 | 1.07 | 3 | 3.78 | 79 | 1.38 | 11 | 10.01 | 110 | 98 |
| Pneumonia (480–486) | 26 | 44.22 | 59** | 46 | 48.89 | 94 | 0.61* | 45 | 54.26 | 83 | 0.75 | 117 | 147.41 | 79** | 96 |
| Bronchitis (460–466, 490–493) | 42 | 59.01 | 71** | 42 | 62.47 | 67** | 0.93 | 45 | 52.64 | 85 | 0.78 | 129 | 174.18 | 74*** | 74*** |
| Diseases of digestive system (520–577) | 23 | 36.79 | 63** | 40 | 38.10 | 105 | 0.60* | 26 | 34.16 | 76 | 1.36 | 89 | 109.09 | 82* | |
| Peptic ulcer (531–534) | 10 | 10.84 | 92 | 10 | 11.36 | 88 | 1.07 | 4 | 10.03 | 40 | 1.85 | 24 | 32.25 | 74 | 68* |
| Cirrhosis (571) | 3 | 8.68 | 35** | 8 | 8.58 | 93 | 0.34 | 4 | 6.13 | 65 | 0.75 | 15 | 23.40 | 64 | 64* |
| Diseases of genitourinary system (580–629) | 18 | 15.32 | 117 | 14 | 16.84 | 83 | 1.33 | 13 | 17.15 | 76 | 1.12 | 45 | 49.33 | 91 | |
| Prostatic hyperplasia (600) | 6 | 2.06 | 291* | 2 | 2.37 | 84 | 2.72 | 2 | 2.36 | 85 | 1.52 | 100 | 6.79 | 147 | 130 |
| Diseases of pregnancy (630–678) | 0 | 0.06 | 0 | 0 | 0.16 | 0 | – | 3 | 0.82 | 368 | 0.00 | 3 | 1.04 | 288 | |
| Symptoms, signs and ill-defined conditions (780–796) | 1 | 1.76 | 57 | 4 | 2.04 | 196 | 0.27 | 8 | 2.67 | 299* | 0.20* | 13 | 6.47 | 201* | |
| Accidents and violence (800–999) | 51 | 67.12 | 76* | 64 | 75.25 | 85 | 0.89 | 66 | 50.70 | 130* | 0.60** | 181 | 193.21 | 94 | 91 |
| All causes other than cancer | 961 | 987.24 | 97 | 983 | 1012.44 | 97 | 0.98 | 873 | 856.00 | 102 | 0.92 | 2817 | 2856.67 | 99 | 91 |

$^*P < 0.05$; $^{**}P < 0.01$; $^{***}P < 0.001$.

a range of half times (ICRP, 1986). The actual solubility class was not known for each plutonium worker and most may have been exposed to a mixture of the two solubility classes of plutonium. We conducted separate analyses of doses for these organs assuming lung clearance rates of either weeks or years. However, we present analyses based on the Y class because measurements made on the solubility of plutonium compounds commonly found at Sellafield show that the majority exhibit behaviour closest to class Y (Riddell et al, 1998). Plutonium alpha and $^{241}$Pu were combined for the assessment of organ-specific doses. Table 2 shows the collective whole-body doses accumulated up to 1990 from external radiation for all radiation workers and for plutonium workers, and also shows the estimated organ-specific and 'effective' whole-body doses from plutonium exposure for those workers for whom such an assessment was made.

Figure 1 shows the number of plutonium workers by year from 1951, when plutonium exposure monitoring was started at the plant, until 1990. The numbers rose up until 1962 to about 2300 workers and, thereafter, stayed between about 1900 and 2300 workers per year up to 1979. Note that the decline in more recent years arises, at least in part, because we have only included workers in the study who were first employed before 1976. Also shown in Figure 1 are the estimated average annual uptakes of $^{241}$Pu and plutonium alpha for the workers for whom such assessments were

*© Cancer Research Campaign 1999* 

**Table 6** Deaths from selected cancers among radiation workers by cumulative external radiation dose (adjusted for age, sex and calendar period). Figures in parentheses are expected distribution of deaths assuming no relation between dose and cancer risk. Data in the body of the table are for analyses including no lag period

| Cancer site (ICD code – eighth revision) | External radiation dose monitored (mSv) | | | | | | | Total deaths | z-statistic[a] by lag of radiation dose (years) | | |
|---|---|---|---|---|---|---|---|---|---|---|---|
| | <10 | 10–19 | 20–49 | 50–99 | 100–199 | 200–399 | 400+ | | 0 | 10 | 20 |
| Oesophagus (150) | 3 (4.0) | 0 (2.6) | 5 (5.7) | 9 (4.7) | 1 (4.6) | 7 (4.5) | 5 (3.9) | 30 | 1.04 | 0.82 | 0.32 |
| Stomach (151) | 14 (12.6) | 8 (6.9) | 14 (14.2) | 12 (12.8) | 8 (11.9) | 11 (10.8) | 11 (8.8) | 78 | 0.48 | 0.22 | –0.51 |
| Colon (153) | 11 (8.4) | 6 (4.4) | 5 (9.3) | 10 (8.3) | 8 (7.8) | 8 (7.5) | 4 (6.3) | 52 | –0.91 | –0.48 | –0.73 |
| Rectum (154) | 4 (5.7) | 2 (2.5) | 5 (5.4) | 4 (4.9) | 6 (4.9) | 6 (4.5) | 5 (4.1) | 32 | 0.99 | 1.27 | 0.98 |
| Pancreas (157) | 5 (4.4) | 4 (3.0) | 4 (5.8) | 6 (4.5) | 3 (4.8) | 4 (5.0) | 6 (4.5) | 32 | 0.38 | 0.56 | 1.57 |
| Lung (162) | 39 (39.2) | 19 (21.5) | 51 (44.0) | 38 (37.4) | 36 (36.3) | 42 (37.0) | 21 (30.4) | 246 | –1.43 | –0.78 | –1.27 |
| Pleura (163.0) | 2 (1.8) | 1 (1.3) | 1 (2.4) | 1 (2.0) | 3 (2.1) | 5 (2.3) | 1 (2.1) | 14 | 0.14 | 0.28 | –0.60 |
| Melanoma (172) | 0 (1.0) | 1 (0.5) | 1 (0.9) | 1 (0.8) | 2 (0.8) | 0 (0.7) | 0 (0.5) | 5 | –0.72 | –0.60 | –0.33 |
| Breast (174) | 3 (4.2) | 1 (1.4) | 1 (1.6) | 3 (0.5) | 0 (0.3) | 0 (0.0) | 0 (0.0) | 8 | 1.00 | 0.73 | 1.48 |
| Prostate (185) | 5 (7.0) | 3 (3.6) | 9 (7.7) | 8 (6.5) | 8 (6.2) | 8 (6.9) | 3 (6.2) | 44 | –0.98 | –0.77 | 0.02 |
| Bladder (188) | 3 (3.8) | 5 (2.3) | 5 (4.8) | 2 (4.2) | 4 (4.5) | 6 (5.2) | 4 (4.4) | 29 | –0.16 | 0.31 | –0.70 |
| Kidney (189.0) | 1 (1.7) | 2 (1.1) | 1 (2.4) | 7 (1.9) | 1 (2.0) | 1 (2.2) | 0 (1.8) | 13 | –1.76[b] | –1.68[b] | –1.56[b] |
| Brain and CNS (191–192) | 1 (3.2) | 3 (2.0) | 5 (3.6) | 4 (2.5) | 2 (2.6) | 2 (2.3) | 1 (1.7) | 18 | –0.62 | –0.48 | 0.15 |
| Ill-defined and secondary (195–199) | 5 (7.7) | 6 (4.4) | 7 (8.6) | 4 (6.7) | 9 (6.2) | 8 (6.2) | 6 (5.2) | 45 | 0.94 | 1.72* | 1.34 |
| Non-Hodgkin's lymphoma (200, 202) | 5 (2.8) | 2 (1.9) | 3 (3.5) | 1 (3.0) | 5 (3.1) | 1 (3.0) | 3 (2.7) | 20 | –0.30 | –0.04 | 0.74 |
| Hodgkin's disease (201) | 1 (0.8) | 1 (0.6) | 1 (1.0) | 0 (0.9) | 2 (0.7) | 0 (0.6) | 0 (0.4) | 5 | –0.80 | –0.37 | 0.49 |
| Multiple myeloma (203) | 0 (1.3) | 0 (0.8) | 2 (1.5) | 3 (1.2) | 1 (1.1) | 0 (1.1) | 2 (1.0) | 8 | 0.80 | 1.44 | 2.53[b] |
| Leukaemia (204–208) | 4 (2.8) | 1 (1.7) | 1 (3.1) | 2 (2.5) | 2 (2.4) | 2 (2.0) | 4 (1.5) | 16 | 2.03[b] | 0.44 | 0.64 |
| | | | | | | | | | 2 year lag = 1.73[b] | | |
| Leukaemia (excluding chronic lymphatic) (204.0, 204.2–208.9) | 2 (2.3) | 1 (1.3) | 1 (2.5) | 1 (2.0) | 2 (2.1) | 2 (1.6) | 4 (1.2) | 13 | 2.77[b] | 1.00 | 1.36 |
| | | | | | | | | | 2 year lag = 2.43[b] | | |
| All lymphatic and haematopoietic (200–209) | 10 (7.8) | 4 (5.1) | 7 (9.3) | 7 (7.7) | 10 (7.5) | 3 (6.9) | 9 (5.7) | 50 | 0.88 | 0.63 | 1.94* |
| All neoplasms (140–209) | 117 (121.7) | 71 (66.3) | 130 (134.1) | 119 (111.1) | 106 (107.7) | 116 (107.4) | 79 (89.7) | 738 | –0.76 | 0.07 | –0.10 |
| All causes of death | 464 (447.6) | 248 (242.3) | 470 (497.7) | 417 (414.2) | 375 (391.2) | 413 (380.6) | 295 (308.4) | 2682 | –0.20 | 1.37 | 2.42** |
| Person–years at risk | 67 518 | 35 473 | 59 731 | 42 901 | 38 275 | 28 808 | 16 664 | 289 370 | | | |

[a]Absolute values of the z-statistic corresponding (approximately) to *P*-values 0.05, 0.01 and 0.001 are 1.64, 2.33 and 3.09 respectively. [b]*P*-values computed by simulation: kidney (0-year lag = 0.027; 10-year lag = 0.030; 20-year lag = 0.039), myeloma (20-year lag = 0.017), leukaemia (0-year lag = 0.027; 2-year lag = 0.050), leukaemia excluding CLL (0-year lag = 0.006; 2-year lag = 0.015). *$P < 0.05$; **$P < 0.01$.

made (see above). Uptake of plutonium alpha was greatest in the early years of the plant's operation and declined markedly thereafter. Estimated uptake of $^{241}$Pu rose from 1960 to a maximum around 1971 and has declined since then. Figure 2 shows the distributions of estimated total uptakes of plutonium alpha and $^{241}$Pu among the plutonium workers for whom uptakes were assessed.

We derived an estimate of the 'effective' whole-body plutonium dose (mSv) from the organ-specific doses supplied by BNFL. This was used to assess mortality in relation to plutonium exposure for all cancers. The estimate of the effective whole-body dose was based on the method detailed in ICRP 30 (ICRP, 1979) and calculated as the weighted sum of organ-specific doses. The weighting factor for a particular organ represents the contribution of that organ to the total stochastic risk (for both fatal malignant and hereditary disease) due to the uniform irradiation of the whole body. Using the ICRP 30 model, the weighting factor for gonads is large (25%), because of the genetic risk, and was considered to be inappropriate for the purposes of this study. Thus, in estimating effective whole-body dose for these analyses, we used revised weighting factors derived by excluding gonads and scaling up the weights associated with the other organs proportionately. The weights applied were thus: bone marrow 0.16; bone surface 0.04; lung 0.16; breast (soft tissue) 0.20; thyroid (soft tissue) 0.04; stomach 0.08; small intestines 0.08; upper large intestines 0.08; lower large intestine 0.08; and liver 0.08. Effective whole-body doses were calculated assuming both weekly and yearly lung clearance rates.

### Statistical analyses

The mortality rates of workers in Sellafield were compared with those of the population of England and Wales and also with the rates for Cumbria. Cancer incidence rates were compared with those of England and Wales and of the Northern UK region. The expected number of deaths or cancer registrations for each cause were estimated by multiplying the number of person–years at risk during the study period by the appropriate national, Northern UK or Cumbrian rates. Each worker contributed person–years at risk

 *© Cancer Research Campaign 1999*

**Table 7** Deaths from selected cancers among plutonium workers by cumulative organ-specific plutonium dose plus external radiation dose, assuming yearly (Y) clearance (adjusted for age, sex and calendar period). Figures in parentheses are expected distribution of deaths assuming no relation between dose and cancer risk. Data in the body of the table are for analyses including no lag period

| Cancer site (ICD code – 8th revision) | Plutonium dose plus external dose (mSv) | | | | | | | Total deaths | Z-statistic[a] by lag of radiation dose (years) | | |
|---|---|---|---|---|---|---|---|---|---|---|---|
| | < 10 | 10–19 | 20–49 | 50–99 | 100–199 | 200–399 | 400+ | | 0 | 10 | 20 |
| Stomach (151) | 1 (1.0) | 1 (0.8) | 4 (2.9) | 2 (4.0) | 4 (5.6) | 6 (5.8) | 8 (6.0) | 26 | 0.92 | 0.78 | 0.13 |
| Colon[b] (153) | 1 (0.9) | 0 (0.5) | 2 (2.1) | 3 (2.3) | 5 (3.4) | 3 (3.5) | 2 (3.4) | 16 | –0.85 | –0.48 | 0.14 |
| Pancreas[c] (157) | 0 (0.4) | 1 (0.5) | 1 (2.0) | 3 (1.8) | 2 (2.9) | 4 (4.0) | 5 (4.3) | 16 | 0.36 | 0.17 | 1.35 |
| Lung (162) | 0 (0.7) | 1 (1.1) | 3 (3.4) | 7 (6.0) | 10 (12.9) | 29 (22.9) | 55 (58.0) | 105 | –0.30 | –0.11 | –0.59 |
| Pleura[c] (163) | 0 (0.1) | 0 (0.2) | 0 (0.8) | 1 (0.9) | 2 (1.3) | 3 (1.8) | 1 (1.9) | 7 | –0.21 | 0.03 | –0.86 |
| Breast[c] (174) | 2 (0.4) | 0 (0.6) | 1 (2.7) | 2 (0.9) | 0 (0.3) | 0 (0.1) | 0 (0.0) | 5 | –0.55 | –0.12 | 0.59 |
| Prostate[c] (185) | 0 (0.4) | 0 (0.4) | 3 (1.8) | 4 (2.2) | 3 (3.1) | 3 (3.9) | 3 (4.3) | 16 | –1.02 | –0.75 | –0.62 |
| Bladder[c] (188) | 0 (0.2) | 1 (0.2) | 1 (1.0) | 0 (1.4) | 2 (1.9) | 3 (2.6) | 3 (2.6) | 10 | 0.41 | 0.88 | –0.73 |
| Brain and CNS[c] (191–192) | 0 (0.4) | 0 (0.4) | 1 (1.2) | 3 (1.2) | 1 (1.6) | 1 (1.8) | 2 (1.3) | 8 | 0.39 | 0.12 | 1.16 |
| Ill-defined and secondary[c] (195–199) | 2 (1.2) | 1 (0.8) | 0 (2.9) | 2 (2.8) | 6 (4.1) | 6 (4.4) | 4 (4.8) | 21 | 0.11 | 0.47 | –0.12 |
| Non-Hodgkin's lymphoma[c] (200, 202) | 2 (0.3) | 0 (0.4) | 2 (1.4) | 1 (1.5) | 2 (2.1) | 2 (2.6) | 2 (2.6) | 11 | –0.82 | –0.49 | –0.12 |
| Leukaemia[d] (204–208) | 0 (0.1) | 0 (0.2) | 0 (0.6) | 1 (0.8) | 1 (1.5) | 2 (1.8) | 3 (2.0) | 7 | 1.11<br>2-year lag 1.25 | –0.07 | 0.30 |
| Leukaemia[d] (excluding chronic lymphatic) (204.0, 202.2–208.9) | 0 (0.1) | 0 (0.2) | 0 (0.4) | 1 (0.6) | 0 (1.2) | 2 (1.6) | 3 (1.8) | 6 | 1.31<br>2-year lag 1.44 | –0.02 | 0.37 |
| All malignant neoplasms[e] | 6 (5.3) | 9 (4.4) | 16 (19.2) | 25 (28.2) | 55 (53.5) | 103 (85.4) | 86 (104.0) | 300 | –1.72* | –1.33 | –1.02 |

[a]Absolute values of the *z*-statistic corresponding (approximately) to *P*-values 0.05, 0.01 and 0.001 are 1.64, 2.33 and 3.09 respectively. [b]Trend calculated using lower large intestine dose. [c]Trend calculated using soft tissue dose. [d]Trend calculated using red bone marrow dose. [e]Trend calculated using estimated 'effective' whole body dose (see text). *$P < 0.05$

from the date of first employment at Sellafield, or from 1 January 1947 for three workers with earlier employment dates, through to the earliest of 31 December 1992, the date of emigration, death or when last traced alive. For the analysis of cancer incidence, workers contributed person–years from the latest of their date of first employment or 1 January 1971 to the earliest of 31 December 1986 or the date of emigration, death or when last traced alive. Mortality and cancer incidence were also compared between radiation workers and non-radiation workers and between workers monitored for plutonium exposure and other radiation workers. For these comparisons, we used maximum likelihood estimates of rate ratios and likelihood ratio tests based on Poisson models.

The risk of cause-specific mortality was investigated in relation to cumulative external radiation exposure and cumulative plutonium doses. These were analysed by comparing death rates among workers who had accumulated different levels of exposure and performing a test for trend to assess the statistical significance of any association. In general, for the trend tests, we used the dose categories used in previous analyses (Douglas et al, 1994) (as shown in Table 6). However, the plutonium doses to organs other than the lungs, bone surfaces, liver and red bone marrow were relatively low (Table 2) and for analyses of cancers of these organs, for plutonium dose alone, we used the dose categories <2, 2–7.9 and ≥ 8 mSv for trend tests. The risks of cancer of the testes, bone, liver and small intestines in relation to the relevant organ-specific plutonium doses were not assessed because of the small number of deaths observed from these causes. Similar dose–response analyses were conducted for cancer registrations. The trend analyses for radiation doses from plutonium were based on 4609 workers who had uptake assessments.

Nearly all of the plutonium workers also had external radiation recorded. Analyses combining plutonium doses and external doses were conducted by adding organ-specific or whole-body plutonium doses to the whole-body external radiation dose.

To allow for any delayed effect of radiation exposure, trend analyses were lagged by 0, 2, 10 and 20 years for leukaemia and by 0, 10, 20 years for all other cancers. All analyses were adjusted by age, sex, calendar period and industrial status (Smith and Douglas, 1986) through stratification.

One-sided statistical significance tests were used because our prior hypothesis was that workers who had been exposed to higher levels of radiation would be expected to show increased cancer death and registration rates. For consistency, unless otherwise stated, all significance tests presented are one-sided in the direction of the observed difference or trend. If the number of deaths or cancer registrations was less than 20, the statistical significance for a rate ratio and trend statistic was checked by simulation.

### RESULTS

### Mortality

Table 3 shows standardized mortality ratios (SMRs) for plutonium workers, all radiation workers and all workers, based on comparison with the mortality rates of the general population of England and Wales and of Cumbria, for all causes of death and all cancers. In general, and as found in previous analyses (Smith and Douglas, 1986; Douglas et al, 1994), the overall mortality rate and the cancer mortality rate of the Sellafield workers were close to those of the general population of England and Wales (SMRs 98 and 95 respectively). Mortality rates from cancer for all radiation workers were 4% less than those for England and Wales, and those for plutonium workers were very close to those for England and Wales

© Cancer Research Campaign 1999 

**Table 8** Observed and expected numbers of cancer registrations from cancer-specific sites among plutonium and other radiation workers and rate ratios comparing plutonium workers with other radiation workers

| Cancer site (ICD code – eighth revision) | Plutonium workers | | | Other radiation workers | | | Rate ratio (Plutonium vs other rad.) |
|---|---|---|---|---|---|---|---|
| | Obs | Exp E&W | SRR E&W | Obs | Exp E&W | SRR E&W | |
| Lip (140) | 2 | 0.88 | 227 | 0 | 0.79 | 0 | ∞ |
| Tongue (141) | 2 | 1.20 | 166 | 3 | 1.08 | 278 | 0.55 |
| Mouth and pharynx (143–149) | 0 | 3.86 | 0 | 3 | 3.47 | 87 | 0.00 |
| Oesophagus (150) | 11 | 6.84 | 161 | 11 | 6.17 | 178 | 0.86 |
| Stomach (151) | 17 | 20.95 | 81 | 17 | 19.03 | 89 | 0.82 |
| Small intestines (152) | 2 | 0.60 | 334 | 0 | 0.54 | 0 | ∞ |
| Colon (153) | 19 | 18.59 | 102 | 20 | 17.37 | 115 | 0.84 |
| Rectum (154) | 18 | 15.62 | 115 | 14 | 14.26 | 98 | 1.23 |
| Liver and gall bladder (155–156) | 0 | 3.13 | 0 | 0 | 2.87 | 0 | – |
| Pancreas (157) | 11 | 8.85 | 124 | 6 | 8.08 | 74 | 1.43 |
| Larynx (161) | 3 | 5.26 | 57 | 3 | 4.60 | 65 | 0.92 |
| Lung (162) | 81 | 85.53 | 95 | 59 | 76.01 | 78* | 1.18 |
| Pleura (163.0) | 1 | 1.15 | 87 | 3 | 1.00 | 299 | 0.26 |
| Bone (170) | 0 | 0.66 | 0 | 1 | 0.60 | 166 | 0.00 |
| Connective tissue (171) | 2 | 1.47 | 136 | 4 | 1.37 | 292 | 0.46 |
| Melanoma (172) | 1 | 2.96 | 34 | 4 | 2.95 | 136 | 0.29 |
| Breast (174) | 4 | 3.32 | 121 | 4 | 6.84 | 58 | 3.81 |
| Uterus (180–182) | 0 | 1.00 | 0 | 2 | 2.34 | 86 | 0.00 |
| Ovary (183) | 1 | 0.50 | 200 | 2 | 1.16 | 172 | 1.46 |
| Other female genital (184) | 0 | 0.04 | 0 | 0 | 0.10 | 0 | – |
| Prostate (185) | 16 | 18.36 | 87 | 15 | 17.01 | 88 | 1.00 |
| Testis (186) | 1 | 2.91 | 34 | 4 | 2.69 | 149 | 0.21 |
| Other male genital (172.5, 187) | 0 | 0.98 | 0 | 0 | 0.87 | 0 | – |
| Bladder (188) | 16 | 19.27 | 83 | 13 | 17.32 | 75 | 1.07 |
| Kidney (189) | 3 | 5.39 | 56 | 3 | 4.83 | 62 | 0.93 |
| Brain and CNS (191–192) | 6 | 6.11 | 98 | 4 | 5.54 | 72 | 1.35 |
| Thyroid (193) | 0 | 0.80 | 0 | 3 | 0.81 | 371* | 0.00 |
| Ill-defined and secondary (195–199) | 17 | 13.28 | 128 | 17 | 12.20 | 139 | 0.90 |
| Non-Hodgkin's lymphoma (200, 202) | 5 | 6.76 | 74 | 2 | 6.22 | 32* | 2.08 |
| Hodgkin's disease (201) | 6 | 2.76 | 217 | 3 | 2.57 | 117 | 1.65 |
| Multiple myeloma (203) | 3 | 3.18 | 94 | 1 | 2.90 | 34 | 3.01 |
| Leukaemia (204–208) | 3 | 7.51 | 40* | 10 | 6.93 | 144 | 0.31* |
| Leukaemia excluding CLL (204.0, 204.2–208.9) | 3 | 5.20 | 58 | 6 | 4.81 | 125 | 0.50 |
| Other neoplasms (140–209 excluding above) | 3 | 6.55 | 46 | 4 | 6.08 | 66 | 0.61 |
| All neoplasms (excluding skin) | 254 | 276.3 | 92 | 235 | 256.6 | 92 | 0.99 |
| Person–years at risk | | 69 472.1 | | | 66 629.9 | | |

**P* < 0.05; ***P* < 0.01; ****P* < 0.001.

(SMR = 100). The findings with respect to cancer were little changed if comparison was made with mortality rates for Cumbria, rather than mortality rates for England and Wales. However, whereas the mortality rates of Sellafield workers from all causes other than cancer were similar to those of England and Wales, they were significantly lower than those of the population of Cumbria.

SMRs for all workers (based on mortality rates for England and Wales) for all causes of death and all cancers were examined according to sex, industrial status, calendar period, age at death, time since first employment and duration of employment at the plant. Results were broadly similar to those reported previously (Smith and Douglas, 1986; Douglas et al, 1994) and are not described here.

Table 4 shows SMRs for all workers, plutonium workers and other radiation workers for specific cancer sites. Also shown are rate ratios comparing mortality among plutonium workers with other radiation workers, and comparing radiation workers to non-radiation workers.

Comparing death rates in the total work force with those of the population of England and Wales, there were significant excesses of deaths from cancers of the pleura (14 observed vs. 4.0 expected, < 0.001) and thyroid (6 vs. 2.2, $P = 0.02$) and significant deficits of deaths from cancers of the mouth and pharynx (6 vs. 10.8, $P = 0.04$), liver and gallbladder (4 vs 14.4, $P < 0.001$), larynx (5 vs. 9.5, $P = 0.04$), lung (338 vs. 381.3, $P = 0.01$) and leukaemia (20 vs. 28.1, $P = 0.04$). Cumbrian rates were available for lung cancer and leukaemia and the deficits of deaths from these cancers were not significant when compared with these rates. The excesses of deaths from cancers of the pleura and thyroid were found in our previous analysis (Douglas et al, 1994). Since that time, there have been five additional deaths from pleural cancer (two in 1989, one in 1991 and two in 1992), but none from thyroid cancer. All 14 deaths from pleural cancer occurred among radiation workers, but the number expected among non-radiation workers was small (0.8). The difference in risk between radiation and non-radiation workers was just significant ($P = 0.05$). The increased risk was of similar magnitude among plutonium workers and other radiation workers. The previously reported excess of cancers of ill-defined and secondary sites (Smith and Douglas, 1986) was now no longer statistically

**Table 9** Cancer registrations among plutonium workers by cumulative organ-specific plutonium dose plus external radiation dose, assuming yearly (Y) clearance (adjusted for age, sex and calendar period). Figures in parentheses are expected distribution of cancer registrations assuming no relation between dose and cancer risk. Data in the body of the table are for analyses including no lag period

| Cancer site (ICD code – 8th revision) | Plutonium dose plus external dose (mSv) | | | | | | | Total cancer registrations | Z-statistic[a] by lag of radiation dose (years) | | |
|---|---|---|---|---|---|---|---|---|---|---|---|
| | <10 | 10–19 | 20–49 | 50–99 | 100–199 | 200–399 | 400+ | | 0 | 10 | 20 |
| Stomach (151) | 1 (0.6) | 1 (0.4) | 1 (1.6) | 2 (2.0) | 3 (3.1) | 4 (3.1) | 2 (3.2) | 14 | –0.62 | –0.84 | –1.58 |
| Colon (153)[c] | 0 (0.6) | 0 (0.4) | 1 (1.8) | 3 (2.1) | 2 (2.8) | 4 (3.3) | 4 (3.1) | 14 | 0.90 | 1.23 | 1.20 |
| Pancreas[d] (157) | 0 (0.3) | 1 (0.3) | 0 (1.2) | 3 (1.2) | 0 (1.8) | 2 (2.7) | 4 (2.6) | 10 | 0.74 | 0.42 | 0.63 |
| Lung (162) | 0 (0.5) | 1 (0.8) | 3 (2.4) | 4 (4.0) | 4 (8.5) | 17 (15.5) | 41 (38.4) | 70 | 0.76 | 0.69 | 0.08 |
| Prostate[d] (185) | 0 (0.4) | 0 (0.3) | 1 (1.1) | 4 (1.5) | 2 (2.3) | 2 (2.8) | 2 (2.8) | 11 | –0.78 | –0.32 | –0.95 |
| Bladder[d] (188) | 0 (0.3) | 1 (0.4) | 0 (1.2) | 0 (1.6) | 2 (2.0) | 5 (2.9) | 3 (2.6) | 11 | 0.96 | 0.22 | –0.99 |
| Brain and CNS[d] (191–192) | 0 (0.3) | 0 (0.3) | 1 (0.8) | 2 (0.7) | 0 (1.1) | 0 (1.0) | 2 (0.8) | 5 | 0.85 | –0.20 | 0.51 |
| Ill-defined and secondary[d] (195–199) | 2 (1.3) | 0 (0.7) | 0 (1.8) | 2 (2.0) | 6 (2.7) | 2 (2.3) | 1 (2.2) | 13 | –0.59 | 0.07 | –0.40 |
| All lymphatic and haematopoietic[e] (200–209) | 1 (1.4) | 0 (0.6) | 3 (1.5) | 1 (1.7) | 0 (2.6) | 3 (3.4) | 7 (3.8) | 15 | 1.95[b] | 1.74[b] | 3.78[b] |

[a]Absolute values of the z-statistic corresponding (approximately) to *P*-values 0.05, 0.01 and 0.001 are 1.64, 2.33 and 3.09 respectively. [b]*P*-values computed by simulation: all lymphatic and haematopoietic neoplasms (0-year lag = 0.024; 10-year lag = 0.046; 20-year lag <0.001). [c]Trends were calculated using lower large intestine dose. [d]Trends were calculated using soft tissue dose. [e]Trends were calculated using red bone marrow dose.

significant. The sites with a deficit of deaths are similar to those previously reported, but, at that time, the findings for cancers of the mouth and pharynx and for leukaemia were not statistically significant.

The only cancer sites for which there was a significant difference in the rates among radiation and non-radiation workers were for cancer of the pleura and cancer of the prostate (rate ratio 2.2, $P = 0.05$), the latter being due mainly to a deficit of deaths among non-radiation workers (compared with mortality rates for England and Wales).

Comparing plutonium workers with other radiation workers, the overall death rates from cancer were similar (rate ratio 1.05) but there were significant excesses of deaths among the former group from cancer of the breast (rate ratio 7.7, $P = 0.01$) and cancers of ill-defined and secondary sites (rate ratio 1.9, $P = 0.04$). For breast cancer, there was a significant excess among plutonium workers and a significant deficit among other radiation workers, compared with the rates for England and Wales.

Mortality from causes other than cancer for the different worker groups is shown in Table 5. The only causes of death for which there were significant excesses of deaths among all workers, compared with mortality rates for England and Wales, were for diseases of the circulatory system, owing to an excess of deaths from ischaemic heart disease (1354 vs. 1217.7, $P < 0.001$), and deaths from ill-defined conditions (13 vs. 6.5, $P = 0.02$). The former excess was not apparent when expected numbers were based on Cumbrian mortality rates. Compared with the rates for England and Wales, there were significant deficits of deaths from infectious and parasitic diseases, tuberculosis, diseases of nervous and sense organs, diseases of the respiratory system (pneumonia and bronchitis) and diseases of the digestive system and also of diabetes, cerebrovascular diseases, peptic ulcer and cirrhosis when compared with Cumbrian death rates.

The only cause of death for which there was a significant excess of deaths among radiation workers compared with non-radiation workers was for cerebrovascular diseases. There were significant deficits of deaths among radiation workers compared with other workers from tuberculosis, diseases of the respiratory system, ill-defined conditions and accidents and violence.

There were significant deficits of deaths due to pneumonia and to diseases of the digestive system among plutonium workers compared with other radiation workers and a significant excess of deaths due to cerebrovascular diseases. For no other cause of death was there a significant difference in the mortality rates of plutonium workers compared with other radiation workers.

### Trends of mortality with cumulative external radiation dose

The results for trend analyses examining associations between cumulative radiation dose and the risk of death from cancer are shown in Table 6. Only those sites for which there were five or more deaths are included. Significant positive trends were seen for ill-defined and secondary cancers (with a lag of 10 years, $P = 0.04$), leukaemia (with no lag, simulated $P = 0.03$; 2-year lag, simulated $P = 0.05$), myeloma (with a lag of 20 years, $P = 0.02$) and all lymphatic and haematopoietic cancers (with a lag of 20 years, $P = 0.03$). Excluding the three deaths from chronic lymphatic leukaemia strengthened the leukaemia trend (with no lag, simulated $P = 0.006$; 2-year lag, simulated $P = 0.02$). There was also a significant positive trend for all causes of death with a lag of 20 years ($P = 0.008$). This was due mainly to significant trends for ischaemic heart disease, mental disorders and pneumonia (data not shown). There was a significant negative association between mortality from cancer of the kidney and cumulative dose with no lag period (simulated $P = 0.03$) and lags of 10 years (simulated $P = 0.03$) and 20 years (simulated $P = 0.04$).

### Trends with plutonium dose

Table 7 shows analyses for plutonium workers relating the risk of death from cancers of specific sites to the estimated organ-specific radiation doses from plutonium exposure and external radiation exposure combined. The only statistically significant finding was a negative trend between the risk of death from any cancer and cumulative radiation dose, assuming no lag period. There was also a significant negative trend for all cancers combined (with no lag) assuming plutonium exposure was in the 'W' class (results not

© Cancer Research Campaign 1999

shown). With the same assumption, there were also significant negative associations for deaths from lung cancer with 0-, 10- or 20-year lags.

### Cancer incidence

The period for which cancer registrations were analysed is the same as that considered in our previous report (Smith and Douglas, 1986), though 11 more cancer registrations have been discovered in the interim and one other which had been classified as 'other cancer' was changed to stomach cancer. The overall findings for all workers were essentially the same as reported previously (Douglas et al, 1994) and are not tabulated. The only site for which there was a significant excess of cancer registrations was for cancers of ill-defined and secondary sites (53 observed vs 35.5 expected, $P = 0.004$). There were significant deficits for cancers of the liver and gallbladder (1 vs 8.3), breast (21 vs 40.1) and all sites combined (664 vs 750.0). Compared with Northern region rates, the excess of cancers of ill-defined and secondary sites was not statistically significant, but there were additional significant deficits of registrations from cancers of the mouth and pharynx (5 vs 13.2), stomach (45 vs 62.5), larynx (7 vs 14.3), lung (192 vs 256.0) and bladder (35 vs 50.0).

Table 8 shows the number of cancer registrations for different sites among plutonium workers and among other radiation workers compared with expected numbers based on rates for England and Wales. The incidence rate of all cancers combined was similar among plutonium workers and among other radiation workers (rate ratio = 0.99). Among radiation workers other than plutonium workers, there were significant deficits of registrations from cancers of the lung and non-Hodgkin's lymphoma and an excess of registrations for thyroid cancer. However, leukaemia was the only cancer for which there was a significant difference in registration rates for the two types of workers, due largely to a significant deficit of registrations from this cause among plutonium workers compared with rates for England and Wales.

### Trends with organ-specific doses

Analyses of trends in cancer incidence in relation to cumulative external radiation dose among all radiation workers have been presented previously (for radiation doses lagged by 0 and 10 years and, in addition, by 2 years for leukaemia) (Douglas et al, 1994). Only for leukaemia (excluding chronic lymphatic) was there a significant increase in risk with cumulative dose (for lags of 0 and 2 years). When radiation doses were lagged by 20 years (which was not done in our previous analyses), there were significant positive trends with cumulative radiation dose for cancers of the brain and CNS (simulated $P = 0.03$), non-Hodgkin's lymphoma (simulated $P = 0.03$) and all lymphatic and haematopoietic neoplasms ($P = 0.005$).

Table 9 shows trends in cancer incidence among plutonium workers in relation to cumulative external radiation dose plus the organ-specific plutonium doses. The only statistically significant findings were positive trends with cumulative dose, for all lag periods considered, for all lymphatic and haematopoietic neoplasms (which were considered together because of the small numbers of registrations from cancers of the component sites). Of the seven cancers in the highest exposure group (≥400 mSv), with zero lag, there were two registrations from each of leukaemia, myeloma and Hodgkin's disease and one from non-Hodgkin's lymphoma. This positive association between cumulative dose and these sites was also significant when the plutonium dose was considered alone, due largely to two workers with a cancer of these sites who had accumulated a plutonium dose (red bone marrow) in excess of 400 mSv [Hodgkin's disease (883 mSv, assuming zero lag) and myeloma (1102 mSv)]. The only other significant finding in relation to cumulative plutonium dose and cancers of specific sites was a positive association for cancer of the pancreas, assuming a lag of 10 years.

### DISCUSSION

Workers at the Sellafield plant had a mortality rate from all causes of death that was 2% less than that of the general population of England and Wales and 8% less than the population of Cumbria. Mortality rates from cancer among workers were 5% and 3% less than those of these two populations respectively. Cancer mortality rates among radiation workers were slightly higher than those among non-radiation workers (rate ratio 1.06), but not significantly so (Tables 3 and 4). These findings are similar to those reported previously, but based on a further 4 years of follow-up (Douglas et al, 1994). In other studies of workers in nuclear plants, mortality rates have been reported which were substantially less than those of the general population. We have discussed previously the significance of the apparent absence of a large 'healthy worker' effect among Sellafield workers (Smith and Douglas, 1986; Douglas et al, 1994) and we do not believe that our failure to find such an effect is because of a deleterious effect of radiation exposure.

In previous analyses (Douglas et al, 1994), we noted significant excess risks of cancers of the pleura, thyroid and of ill-defined and secondary sites among Sellafield workers. The excesses for the first two sites persist with further follow-up, but that for the last site is no longer significant (Table 4), though, as previously we found a significant association between the risk of death from this cause and accumulated external radiation dose (Table 6). This finding is consistent with radiation being a cause of these ill-defined and secondary cancers, but does not account for the excess being among non-radiation workers. Mortality rates for these cancers among radiation workers were slightly lower than those among non-radiation workers (rate ratio 0.96, Table 4). An excess of these cancers has not been found in studies of other radiation workers and nor has the association with cumulative radiation dose (Gilbert et al, 1993; Carpenter et al, 1994; Cardis et al, 1995). Thus, our findings are difficult to interpret for this, by definition, ill-defined group of tumours. There have been no additional deaths from thyroid cancer since our last report but a further five deaths from pleural cancer, bringing the total to 14 against only four expected. The mortality from thyroid cancer was higher among radiation workers than among non-radiation workers, but not significantly. All of the deaths from pleural cancer were among radiation workers, and the difference compared with non-radiation workers was just statistically significant ($P = 0.05$, Table 4). For neither site was there a significant association between the risk of the cancer and accumulated external radiation dose (Table 6, data for thyroid cancer not shown). A significant excess of thyroid cancer has not been found in other studies of nuclear workers (Gilbert et al, 1993; Carpenter et al, 1994; Cardis et al, 1995), but an excess was reported in a national study of radiation workers that included workers from Sellafield (Kendall et al, 1992). We commented at length on the excess of cancer of the pleura in our previous report and although

 © Cancer Research Campaign 1999

the possibility of radiation exposure contributing to this excess cannot be excluded, it is plausible that the major part of the excess is due to exposure to asbestos. It is of interest, however, that a significant excess of deaths from pleural cancer has not been found in other studies of nuclear workers in the UK (Carpenter et al, 1994). The excess at Sellafield was of similar magnitude among plutonium workers and among other radiation workers (Table 4).

Leukaemia is the cancer most readily induced by radiation and is the one for which an excess is most likely to be observed among radiation workers. Since our last report, there have been five additional deaths from this cause in the total worker population, which is about the number expected based on rates for England and Wales, but overall there was a significant deficit of deaths from this cause (20 vs 28.1 expected, $P < 0.05$, Table 4). The deficit was most marked among non-radiation workers, but was also present among radiation workers. However, when leukaemia mortality risk was related to accumulated external radiation dose there was a significant dose–response relationship, using lag periods of 0 and 2 years, which was strengthened when deaths from chronic lymphatic leukaemia were excluded (which cause has not been related to radiation exposure in other studies of radiation-exposed populations). It seems likely that this is a real effect of radiation exposure at the plant and the magnitude of the increase in risk with radiation dose is in line with dose–response estimates that have been derived in populations exposed to high doses of radiation, as we have discussed previously (Douglas et al, 1994).

With continued follow-up of the Sellafield population, substantial numbers of workers have now been followed for several decades. Indeed, the average follow-up time is now 29 years. We have, therefore, examined the relationship between cancer risk and accumulated radiation dose, not only lagged by 0 and 10 years (and 2 years for leukaemia), as in previous analyses (Douglas et al, 1994), but also by 20 years to look for possible effects after long induction times. With this long lag period, the only cancer site for which this gave a significant trend of increasing risk with dose was for myeloma ($P = 0.02$, Table 6), apart from all lymphatic and haematopoietic neoplasms which grouping includes myeloma. This is similar to the finding we reported originally (Smith and Douglas, 1986), using a 15-year lag period and we have speculated that this may represent a true radiation effect (Smith and Douglas, 1986). There has been only one additional death from myeloma among radiation workers since our previous 2 analyses (Smith and Douglas, 1986; Douglas et al, 1994) and the total deaths from this cause among all workers and among radiation workers was about the same as the expected numbers (Table 4).

An unexpected finding was the association between the risk of death from all causes combined and accumulated radiation dose, assuming a 20-year lag period. There was no evidence of such an effect for all cancers combined and the association was due mainly to correlations between risk and cumulative dose for mental disorders, ischaemic heart disease and pneumonia. Although there was a significant excess of deaths from ischaemic heart disease in the total work force, compared with rates for England and Wales, there was no excess when comparison was made with Cumbrian rates, and, for each of these three causes, mortality rates among radiation workers were lower than those among non-radiation workers, though in no instance significantly so. There is little evidence from numerous other studies that radiation exposure influences causes of death other than cancer even in populations that have been exposed to relatively high doses, so that our findings are perhaps most likely to be due to chance.

We have not previously examined mortality patterns among plutonium workers at the Sellafield plant and this group is the main focus of this paper. Our analyses are subject to a number of limitations. Plutonium workers were defined as those who had ever provided a urine sample for plutonium assay. Some of these workers will never have been exposed to plutonium and, even for those that were, the exposure may not have been detected because of the low sensitivity of the assay method used, particularly during the early years of the plant's operation. Of the workers for whom a plutonium uptake assessment was made, 21.5% had 'special' assessments of their annual uptakes, taking account not only of urine assays but also knowledge of specific exposure incidents and place of work within the plant. This group included all of those who had been involved in specific exposure incidents or who had been assessed for the nuclear industry compensation scheme. For the remaining workers, annual uptakes were estimated based on urine data using only a mathematical model. There are substantial uncertainties in all these assessments of uptake and few data with which to validate the estimation methods. This is illustrated by the fact that uptake levels were divided by three before they were provided to us by BNFL because of differences between estimates derived from urine assays and levels measured in the small number of workers who have had detailed plutonium burdens measured after death.

As the group of workers for whom special assessments were made included those claiming a compensation award, we were concerned that any systematic difference in the plutonium uptakes estimated by the 'special' and 'standard' methods might lead to bias. We therefore asked that 'standardized' assessments be made for the 644 workers who had a special assessment but who were not known to have had an acute exposure. This comparison showed that estimates of uptakes from standardized assessments were, on average, about 25% higher than those obtained using the special approach. However, there was no evidence that cancer mortality was higher in those having a special assessment. These workers had an SMR of 82 for all cancers (comparing with rates for England and Wales), whereas plutonium workers who had a plutonium dose assessed using the standard method had an SMR of 95. We do not believe, therefore, that the use of the two different dose assessment methods is likely to have seriously biased the analyses.

Uptake assessments were made for 725 workers whose urine assays were all below the limit of detection by setting the last sample at the level of the limit applicable at that time. As well as using the doses based on these uptake assessments, we also conducted statistical analyses in which these workers were assigned zero uptake (and dose). There were no material differences in the two sets of analyses.

There are further uncertainties with respect to dose estimation because the solubility class of plutonium exposure for individual workers at different times was unknown. We therefore assumed both 'W' and 'Y' solubility classes in our analyses – this makes a substantial difference to the estimated lung doses (see Table 2). Many workers probably received a mixture of the two classes of plutonium, though Y-class exposures are likely to have been most common (Riddell et al, 1998). These and other uncertainties complicate considerably our interpretation of analyses relating cancer risk to estimated radiation doses from plutonium exposure.

Workers who had ever been monitored for plutonium exposure had a cancer mortality rate similar to that of the populations of England and Wales (SMR 100) and Cumbria (SMR 101) and a slightly higher rate than other radiation workers (rate ratio 1.05,

© Cancer Research Campaign 1999

Table 3), but this difference was not significantly different. The two groups of workers also had similar cancer incidence rates (rate ratio 0.99, Table 8). Thus, there was no evidence that, overall, the cancer risk among plutonium workers was increased, compared with radiation workers not monitored for exposure to plutonium. Plutonium workers had higher death rates from breast cancer than other radiation workers, the rate among plutonium workers being significantly above that for England and Wales and for other radiation workers being significantly below the national rate (Table 4). Breast cancer incidence rates were also higher among plutonium workers than other radiation workers, though not significantly so (Table 8). All eight deaths from this cancer among radiation workers were in women. When breast cancer risk was related to radiation dose among plutonium workers, there was no evidence of a dose–response relationship (Table 7), which is some evidence against a causative association.

One woman who died of breast cancer had urine samples taken for plutonium monitoring only after the cancer had been diagnosed and all samples were recorded as below the limit of detection. She was not monitored for external radiation exposure and her medical records indicate that the urine samples were taken because of the cancer diagnosis (AE Riddell, personal communication). If this death is excluded from the analyses, the breast cancer mortality rate among plutonium workers is not significantly greater than the England and Wales rate (in Table 4: 5 observed, 2.55 expected) and the excess among plutonium workers compared to other radiation workers is of reduced significance ($P < 0.05$).

The only other cancer site for which plutonium workers had a significantly higher mortality rate than other radiation workers was for cancer of ill-defined and secondary sites (rate ratio 1.9, Table 4), but there was not a relationship between risk and estimated cumulative radiation dose among plutonium workers (Table 7). As discussed above for all radiation workers, this finding is difficult to interpret. With respect to cancer incidence, the overall risk for all cancers combined was similar for plutonium workers and other radiation workers and for no individual site was there a significant difference in the incidence rates.

The absence of any marked difference in the risks of death from causes other than cancer between plutonium workers and other radiation workers (Table 5) is in line with what might be expected on the basis of previous evidence that these causes are not affected by radiation exposure.

Organ-specific annual radiation dose estimates from plutonium exposure were made for specific sites, as listed in Table 2. The estimated collective cumulative doses to bone surfaces, the red bone marrow and the liver were substantial. Those to the lung depended greatly on whether the 'W' or 'Y' solubility class of plutonium was assumed. Estimated doses to the other organs were much lower. Among plutonium workers, we did not find any significant positive trends between cumulative radiation doses to different organs and the risk of death from cancer (Table 6), though there was a significant association with cancer registrations for all lymphatic and haematopoietic cancers combined (Table 9). Wilkinson et al (1987), in a study of plutonium workers at the Rocky Flats weapons facility, reported an excess of lymphopoietic cancers among workers with body burdens of 74 Bq or greater, but the number of cases was small and the excess was not statistically significant.

Taken as a whole, our findings do not suggest that workers at Sellafield who have been exposed to plutonium are at an overall significantly increased risk of cancer, compared either with the population of Cumbria or to other radiation workers at the plant. Compared with the former group, there were excesses of deaths from cancers of the pleura, breast, and ill-defined and secondary sites, but for none of these sites was there evidence that risk was associated with cumulative dose. Because of the indications from a parallel study of an association between the time since first monitoring for plutonium and the risk of deaths from all malignant neoplasms considered (Carpenter et al, 1998), we repeated those analyses using our data for the Sellafield workforce. Comparing plutonium workers with other radiation workers, the ratio of the death rates in the two groups in the periods <10 years, 10–19 years and ≥20 years since monitoring started were 0.82, 1.03 and 1.12. This trend was not statistically significant [$\chi^2$ (1 df trend) = 2.64]. We also found no evidence that deaths from all malignant neoplasms were related to the number of years for which workers had been monitored for plutonium exposure [$\chi^2$ (1 df trend) = 1.27]. In a study of 15 727 men employed at the Los Alamos National Laboratory, Wiggs et al (1994) found that no cause of death was significantly elevated among 303 plutonium-exposed (>74 Bq) workers when compared with other workers. They did observe, however, a non-significant excess of lung cancers among the former group and also a case of osteogenic sarcoma which was in one of the 26 workers on the Manhattan project known to have been exposed to plutonium (Voelz et al, 1997). It is notable that despite the comparatively large radiation doses to the bone surfaces, lungs and liver (Table 2) among Sellafield plutonium workers, no death from bone cancer was observed, the lung cancer mortality rate was similar to that of other radiation workers and non-radiation workers and there was a significant deficit of deaths from liver cancer among plutonium workers (Table 4). The results for lung cancer contrast with those reported from a study among nuclear workers who had been very heavily exposed to plutonium in a nuclear facility in Russia in which a threefold excess of lung cancer was found (147 observed, 45.4 expected) (Koshurnikova et al, 1997).

Our findings with respect to plutonium exposure must be interpreted with caution. Whereas external radiation doses may be estimated relatively well from film badge dosimeters (Kite and Britcher, 1996), those for internal exposures have depended largely on monitoring urine samples and extrapolating to organ–dose estimates based on assays of those samples. There are several important potential sources of error in the dose-estimation process. First, although records were available of urine samples collected for plutonium monitoring, not all workers providing such samples were necessarily exposed to plutonium. The sensitivity of the assay methods to detect and measure plutonium levels varied over time and, in particular, these were relatively poor in the early years of the operation of the plant. Furthermore, calculation of plutonium uptakes based on urine sample assays involves making assumptions about patterns of exposure over time and further assumptions are necessary to convert uptake estimates to organ–dose. It has not been possible to determine the magnitude of the errors that may be inherent in the dose-estimation process, but it is considered that it would not be uncommon for them to be lower by an order of magnitude (A. Riddell, personal communication). This limits the usefulness of quantitative dose–response analyses. The uncertainties with respect to organ doses from plutonium exposure are greatest for workers for whom urine samples were available only prior to 1971. A reflection of the uncertainty is that plutonium uptake estimates for this group of workers originally supplied to us by BNFL were subsequently reduced by a factor of 3 on the basis of an internal BNFL investigation (Riddell

© Cancer Research Campaign 1999

et al, 1998). The revised data were used in the analyses presented. We repeated the analyses shown in Tables 7 and 9 excluding this group of workers (who comprised 55% of all plutonium workers for whom a standardized assessment was made). In general, the results were not notably different from those shown in the tables. The only exception was for cancer registrations from lung cancer, with no lag, for which a significant trend statistic was obtained ($z = 2.25$, $P < 0.05$). This result was based on only 20 lung cancer registrations and no trend was apparent using lags of 10 and 20 years. Furthermore, there was no evidence of a trend with dose, using lags of 0, 10 or 20 years, for lung cancer deaths.

### ACKNOWLEDGEMENTS

This study was supported, in part, by a grant to the London School of Hygiene and Tropical Medicine by British Nuclear Fuels. We are grateful to the many people who helped with the study and advised us. In particular, we thank Allison Douglas, Shahed Murad, Chris Metcalfe, Zongkai Qiao and the staff of the NHS Central Registers. Thanks also are due to an anonymous referee for helpful suggestions. We thank Keith Binks, Tony Riddell, Sheila Jones, Sharon Maxwell and other BNFL staff with whom we liaised to assemble the data for analysis and who provided us with dosimetry data. We are grateful to the Department of Medical Statistics and Evaluation, Imperial College School of Medicine for supporting two of us (RO, JB) during the later stages of the work.

### REFERENCES

Britcher AR, Dalton AP, Riddell AE, Battersby W and Strong R (1994) What do your plutonium in urine results tell you? A tour through 40 years of change at Sellafield. *Radiat Protection Dosimetry* **53**: 1–4

Cardis E, Gilbert ES, Carpenter L, Howe G, Kato I, Armstrong BK, Beral V, Cowper G, Douglas A, Esteve J, Fix J, Fry SA, Kaldor J, Lave C, Smith PG, Voelz G and Wiggs LD (1995) Effects of low doses and low dose-rates of external ionizing radiation: cancer mortality among nuclear industry workers in three countries. *Radiat Res* **142**: 117–132

Carpenter L, Higgins C, Douglas A, Fraser P, Beral V and Smith P (1994) Combined analysis of mortality in three UK nuclear industry workforces, 1946–88. *Radiat Res* **138**: 224–238

Carpenter LM, Higgins CD, Douglas AJ, Maconochie NES, Omar RZ, Fraser P, Beral V and Smith PG (1998) Cancer mortality in relation to monitoring for radionuclide exposure in three UK nuclear industry workforces. *Br J Cancer* **78**: 1224–1232

Clarke RH, Dunster J, Nenot J-C, Smith J and Voelz G (1996) The environmental safety and health risks of plutonium. *J Radiol Protection* **16**: 91–105

Committee on Biological Effects of Ionizing Radiations (1988) *Health Risks of Radon and Other Internally Deposited Alpha-Emitters*. BEIR IV. National Academy Press: Washington DC

Douglas AJ, Omar RZ and Smith PG (1994) Cancer mortality and morbidity among workers at the Sellafield plant of British Nuclear Fuels. *Br J Cancer* **70**: 1232–1243

Gardner MJ, Winter PD, Taylor CP and Acheson ED (1983) *Atlas of Cancer Mortality in England and Wales*. Wiley: Chichester

Gardner MJ, Winter PD and Barker DJP (1984) *Atlas of Mortality from Selected Diseases in England and Wales, 1968–1978*. Wiley: Chichester

Gilbert ES, Cragle DL and Wiggs LD (1993) Updated analyses of combined mortality data for workers at the Hanford site, Oak Ridge National Laboratory, and Rocky Flats nuclear weapons plant. *Radiat Res* **136**: 408–421

International Commission on Radiological Protection (1979) *Limits for Intakes of Radionuclides by Workers*. Publication 30. Pergamon Press: Oxford

International Commission on Radiological Protection (1986) *The Metabolism of Plutonium and Related Elements*, Publication 48. Pergamon Press: Oxford

Jones SR (1985) Derivation and validation of a urinary excretion function for plutonium applicable over tens of years post uptake. *Radiat Protection Dosimetry* **11**: 19–27

Kendall GM, Muirhead CR, MacGibbon BH, O'Hagan JA, Conquest AJ, Goodill AA, Butland BK, Fell TP, Jackson DA, Webb MA, Haylock RGE, Thomas JM and Silk TJ (1992) Mortality and occupational exposure to radiation: first analysis of the National Registry for Radiation Workers. *Br Med J* **304**: 220–225

Kite AV and Britcher AR (1996) Uncertainties in recorded photon radiation doses at Sellafield. *Radiat Protection Dosimetry* **67**: 23–32

Koshurnikova NA, Shilnikova NS, Okatenko PV, Kresolv VV, Bolotnikova MG, Romanov SA and Sokolikov ME (1997) The risk of cancer among nuclear workers at 'Mayak' production asociation: preliminary results of an epidemiological study. In *Implications of New Data on Radiation Cancer Risk*. Boice JD (ed.), pp. 113–122. National Council on Radiation Protection and Measurements: Bethesda MD

Lawson AW, Wraight JC, Wallace B, Bunker A and Strong R (1989) Plutonium deposition in man: comparison between excretion and autopsy studies. In *Radiation Protection – Theory and Practice*. Goldfinch EP ed., pp. 231–234. Institute of Physics Publishing: Bristol

Riddell AE and Britcher AR (1994) Pluto – a software package using the 'maximum likelihood method' to fit plutonium in urine data to an excretion function. *Radiat Protection Dosimetry* **53**: 1–4

Riddell AE, Battersby WP, Britcher AR and Peace MS (1998) The assessment of dose from plutonium for a cancer and morbidity study among Sellafield plutonium workers. *J Radiol Protection* (in press).

Smith PG and Douglas AJ (1986) Mortality of workers at the Sellafield plant of the British Nuclear Fuels. *Br Med J* **293**: 845–854

Voelz GL, Lawrence JNP and Johnson ER (1997) Fifty years of plutonium exposure to the Manhattan project plutonium workers: an update. *Health Phys* **73**: 611–619

Wiggs LD, Johnson ER, Cox-De Vore CA and Voelz GL (1994) Mortality through 1990 among white male workers at the Los Alamos National Laboratory: considering exposures to plutonium and external ionizing radiation. *Health Phys* **67**: 577–588

Wilkinson GS, Tietjen GL, Wiggs LD, Galke WA, Acquavella JF, Reyes M, Voelz GL and Waxweiler RJ (1987) Mortality among plutonium workers at a plutonium weapons facility. *Am J Epidemiol* **125**: 231–250

*© Cancer Research Campaign 1999*
